# Anti-Semaphorin 4D Rescues Motor, Cognitive, and Respiratory Phenotypes in a Rett Syndrome Mouse Model

**DOI:** 10.3390/ijms22179465

**Published:** 2021-08-31

**Authors:** Yilin Mao, Elizabeth E. Evans, Vikas Mishra, Leslie Balch, Allison Eberhardt, Maurice Zauderer, Wendy A. Gold

**Affiliations:** 1 Molecular Neurobiology Research Laboratory, Kids Neuroscience Centre, Kids Research, The Children’s Hospital at Westmead, Westmead, NSW 2145, Australia; matthew.mao@greenlightclinical.com; 2 Discipline of Child and Adolescent Health, Faculty of Medicine and Health, The University of Sydney, Camperdown, NSW 2006, Australia; 3 Vaccinex Inc., Rochester, NY 14620, USA; eevans@vaccinex.com (E.E.E.); vmishra@vaccinex.com (V.M.); LBalch@vaccinex.com (L.B.); awilliams@vaccinex.com (A.E.); mzauderer@vaccinex.com (M.Z.); 4 Molecular Neurobiology Research Laboratory, The Children’s Medical Research Institute, Westmead, NSW 2145, Australia; 5 School of Medical Sciences, Faculty of Medicine and Health, The University of Sydney, Camperdown, NSW 2006, Australia

**Keywords:** Rett syndrome, semaphorin 4D, Plexin B1, monoclonal antibody therapy, behavioural assessments, cytoskeleton, glial cells, neuroinflammation

## Abstract

Rett syndrome is a neurodevelopmental disorder caused by mutations of the methyl-CpG binding protein 2 gene. Abnormal physiological functions of glial cells contribute to pathogenesis of Rett syndrome. Semaphorin 4D (SEMA4D) regulates processes central to neuroinflammation and neurodegeneration including cytoskeletal structures required for process extension, communication, and migration of glial cells. Blocking SEMA4D-induced gliosis may preserve normal glial and neuronal function and rescue neurological dysfunction in Rett syndrome. We evaluated the pre-clinical therapeutic efficacy of an anti-SEMA4D monoclonal antibody in the Rett syndrome Mecp2T158A transgenic mouse model and investigated the contribution of glial cells as a proposed mechanism of action in treated mice and in primary glial cultures isolated from Mecp2T158A/y mutant mice. SEMA4D is upregulated in neurons while glial fibrillary acidic protein and ionized calcium binding adaptor molecule 1-positive cells are upregulated in Mecp2T158A/y mice. Anti-SEMA4D treatment ameliorates Rett syndrome-specific symptoms and improves behavioural functions in both pre-symptomatic and symptomatic cohorts of hemizygous Mecp2T158A/y male mice. Anti-SEMA4D also reduces astrocyte and microglia activation in vivo. In vitro experiments demonstrate an abnormal cytoskeletal structure in mutant astrocytes in the presence of SEMA4D, while anti-SEMA4D antibody treatment blocks SEMA4D–Plexin B1 signaling and mitigates these abnormalities. These results suggest that anti-SEMA4D immunotherapy may be an effective treatment option to alleviate symptoms and improve cognitive and motor function in Rett syndrome.

## 1. Introduction

Rett syndrome is a rare neurodevelopmental disorder, caused by mutations in the X-linked gene encoding methyl-CpG binding protein 2 (*MECP2*). Rett syndrome is the second most common cause of severe intellectual disability after Down syndrome in females, affecting one out of every 10,000 live births [1,2,3]. Girls with Rett syndrome usually appear asymptomatic in their first 6–18 months of life, which is followed by a gradual regressive development with severe motor, cognitive, neurological, and behavioural abnormalities that persist for life [3]. This late onset provides a valuable window of opportunity for therapeutic intervention and the prevention of disease progression.

Reports of altered cytoskeletal structure, morphology, and physiological functions in Rett syndrome astrocytes and microglia suggest the importance of glial cells in the pathogenesis of the disorder. Under pathological conditions or physiological stress, astrocytes and microglia transform from a resting state to an inflammatory state, known as reactive astrocytes and activated microglia [4,5,6]. Reactive astrocytes cause neuronal toxicity by a number of mechanisms, including disruption of glutamate metabolism [7] and microtubule stability [8,9,10], while activated microglia impair neuronal activity in part by releasing excessive glutamate and inflammatory cytokines [11,12].

Semaphorin 4D (SEMA4D), also known as CD100, initially identified as an axonal guidance molecule during neuronal development, has also recently been found to regulate the assembly of the cytoskeleton required for the normal functions and migration of glial precursor cells, which give rise to astrocytes and oligodendrocytes, and activation of microglia [13,14]. SEMA4D plays a critical role in regulating the transition between resting and activated states of astrocytes and microglia, via signalling through its Plexin B1 receptors on glial cells to activate NfκB, a master regulator of inflammatory cytokines [14,15,16,17,18]. Thus, SEMA4D is a unique target of therapeutic development for multiple pathological conditions including Rett syndrome where glial activation is believed to be involved in the pathogenesis of disease.

Anti-SEMA4D monoclonal antibody therapy has been shown to inhibit activation of glial cells and ameliorate neurological disorders in several in vivo experimental models, including a transient middle cerebral artery occlusion mouse model of stroke [19], lysolecithin-induced rat spinal cord lesions, experimental autoimmune encephalomyelitis (EAE) [13], and the YAC128 transgenic model of Huntington’s disease [17]. In addition, pepinemab, a humanised anti-SEMA4D monoclonal antibody, has been evaluated in several clinical trials, including a Phase I trial in patients with multiple sclerosis (NCT01764737) and a Phase II trial in Huntington’s disease (NCT02481674). A Phase I/II study of pepinemab is also planned in Alzheimer’s disease (NCT04381468). Given its significant pharmaceutical potential in several neurological disorders, we explored the therapeutic benefits of anti-SEMA4D antibody in the *Mecp2^T158A^* Rett syndrome mouse model. Here, we demonstrate reversal of key features of the Rett syndrome phenotype following antibody blockade of SEMA4D as well as upregulation of SEMA4D expression during disease progression and the effects of SEMA4D on reactive transformation of mutant glial cells expressing the Plexin B1 receptor. This study expands mechanistic insight into Rett syndrome pathology and suggests the potential of anti-SEMA4D antibody therapy in this paediatric neurological disorder.

## 2. Results

### 2.1. Anti-SEMA4D Therapy Improves Clinical Scores in a Rett Syndrome Mouse Model

Survival profiles of the pre-symptomatic and symptomatic cohorts were analysed and plotted on Kaplan–Meier survival curves (Supplementary Figure S1A,B). There were no significant differences between survival rate in any groups of the 4-week cohort. In the 8-week cohort, there was a significant reduction in survival of *Mecp2^T158A/y^* mice compared to WT mice (*p* = 0.048), but no difference between the treatment groups. All mice were weighed weekly, and their body weights increased over the 10-week trial with no significant differences between groups in the pre-symptomatic and symptomatic cohorts (Supplementary Figure S1C,D).

Both the 4-week cohort (pre-symptomatic) and 8-week (symptomatic) anti-SEMA4D antibody-treated Rett syndrome cohorts showed significant improvements in the phenotypic clinical scores relative to the isotype control antibody groups.

**Figure 1:**
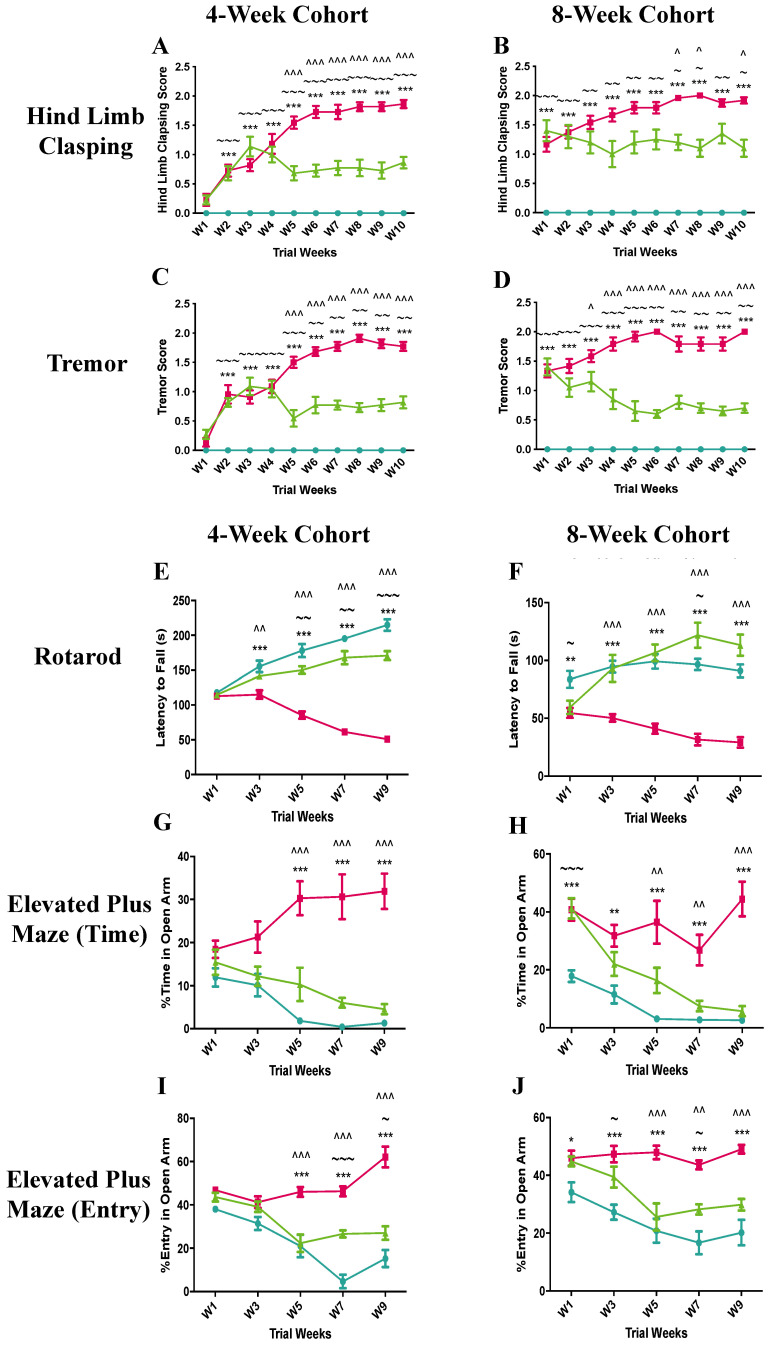
Clinical score assessments and results of rotarod and elevated plus maze tests of 4-week (pre-symptomatic) and 8-week (symptomatic) cohorts in the pre-clinical trial. Data are expressed as mean ± standard error of the mean in hindlimb clasping scores (**A**,**B**), and body tremor scores (**C**,**D**). Latency to fall from the accelerating rotarod apparatus (**E**,**F**), percentage time spent in the open arms of the elevated plus maze apparatus (**G**,**H**), and percentage entry in the open arms of the elevated plus maze apparatus (**I**,**J**) are shown for the 4-week and the 8-week cohorts. 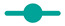 C57BL/6 mice treated with control antibody (WT), 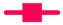 *Mecp2^T158A/y^* mice treated with control antibody (P), 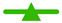 *Mecp2^T158A/y^* mice treated with anti-semaphorin 4D antibody (T). * WT vs. P, ~ WT vs. T, ^ P vs. T. *, ~, and ^ *p* < 0.05; **, ~~, and ^^ *p* < 0.01; ***, ~~~, and ^^^ *p* < 0.001.

Hind limb clasping is a hallmark feature of many Rett syndrome mouse models and a behaviour that is reminiscent of the hand movements seen in Rett syndrome girls. Hind limb clasping in the pre-symptomatic mice that received isotype control antibody progressively worsened throughout the 10-week study, reaching a top mean score of ~1.8 in week 10 (Figure 1A). In contrast, the hind limb clasping score of the anti-SEMA4D antibody-treated *Mecp2^T158A/y^* mice climbed to the maximum of 1.1 in week 3 and was reduced to 0.7 in week 5 where it plateaued for the duration of the study. The hind limb clasping score of the anti-SEMA4D antibody-treated *Mecp2^T158A/y^* mice was significantly lower than that of the *Mecp2^T158A/y^* mice receiving placebo between week 5 and 10 (*p* < 0.001). Similar to the 4-week cohort, the hindlimb clasping in the symptomatic mice that received isotype control antibody progressively worsened throughout the 10-week study, reaching a score of ~1.8 in week 10 (Figure 1B). The hindlimb clasping score of the symptomatic *Mecp2^T158A/y^* mice treated with anti-SEMA4D antibody was significantly lower than that of the *Mecp2^T158A/y^* mice receiving control antibody in week 7, 8, and 10 (*p* < 0.05). The wild-type mice showed no hindlimb clasping throughout the study, as expected.

Tremors are another common feature of Rett syndrome mice models, resulting from a loss of Mecp2 in excitatory neurons. In the pre-symptomatic cohort, the tremor score of *Mecp2^T158A/y^* mice that received isotype antibody control progressively increased to the maximum of 1.8 in week 8, dropping slightly to 1.6 in week 10 (Figure 1C). Tremors in the anti-SEMA4D antibody-treated *Mecp2^T158A/y^* mice progressed at the same rate as the isotype control antibody-treated *Mecp2^T158A/y^* mice for the first four weeks of the trial, after which they dramatically decreased to 0.7 and they remained significantly lower than those of the placebo group for the remainder of the trial (*p* < 0.001). As in the pre-symptomatic cohort, the symptomatic cohort tremor scores in the placebo group progressively increased over the 10-week period and reached a top score of 1.9 in week 10 (Figure 1D). Similarly, the tremors in the anti-SEMA4D antibody treatment group gradually decreased by 50% between week 1 and 5 and then remained constant until the end of the trial. There were statistically significant differences in the tremor score between the treatment and placebo groups from week 3 (*p* < 0.05) to week 10 (*p* < 0.001). The wild-type mice showed no tremors throughout the 10-week trials.

### 2.2. Anti-SEMA4D Therapy Prevents the Onset and Reverses Deficits of Coordination and Cognition

Latency to fall from the acceleratingly rotating rod in the rotarod test was used to assess the coordination function in the treated mice, such that longer latency indicates better coordination function. In the pre-symptomatic cohort, the latency to fall of the *Mecp2^T158A/y^* mice receiving isotype antibody progressively decreased over the 10-week trial and was significantly lower than that of wild-type mice from week 3 onwards (Figure 1E) (*p* < 0.001). The anti-SEMA4D antibody-treated *Mecp2^T158A/y^* mice gradually increased the latency to fall from the rod during the trial, which was significantly higher than that of *Mecp2^T158A/y^* mice receiving isotype control antibody between week 3 (*p* < 0.01) and week 9 (*p* < 0.001). In the symptomatic cohort, the latency to fall in the placebo Rett syndrome group progressively declined during the trial and was significantly lower than that of the wild-type group from week 1 (*p* < 0.01) to week 9 (*p* < 0.001) (Figure 1F). The latency to fall of the *Mecp2^T158A/y^* mice receiving anti-SEMA4D antibody continuously improved over time and was significantly higher than that of *Mecp2^T158A/y^* mice with isotype control antibody from week 3 to week 9 (*p* < 0.001). Surprisingly, the anti-SEMA4D antibody-treated mice appeared to stay significantly longer on the rotating rod compared with the wild-type mice in week 7 (*p* < 0.05).

The elevated plus maze test was employed to evaluate the cognitive functional effects of anti-SEMA4D antibody treatment, based on the general aversion of mice to open spaces. Animals with impaired cognition are less able to recognise the aversive properties of the open arms, detected by spending longer time in the open arms and making more open-arm entries during the test. In the pre-symptomatic cohort, mice from all groups did not demonstrate any changes in cognitive function in the first 3 weeks of the trial (Figure 1G,I). Both the wild-type and anti-SEMA4D antibody-treated *Mecp2^T158A/y^* mice spent less time in the open arms and made fewer open-arm entries over the 10-week pre-clinical trial. In contrast, the *Mecp2^T158A/y^* mice receiving placebo spent more time in the open arms and made more entries into the open arms over the 10-week course. The percentage of time in the open arms and percentage of open-arm entries in the placebo group were significantly higher than those in the other two groups from week 5 to 9 (*p* < 0.001). Similarly, the wild-type and anti-SEMA4D antibody-treated *Mecp2^T158A/y^* mice in the symptomatic cohort spent less time in the open arms and made fewer entries into the open arms over the 10-week trial (Figure 1H,J). The percentage of time spent in the open arms of the *Mecp2^T158A/y^* mice receiving isotype control fluctuated over time and was significantly higher than that of the wild-type mice throughout the trial (*p* < 0.01) and higher than the anti-SEMA4D antibody-treated *Mecp2^T158A/y^* mice between week 5 and week 9 (*p* < 0.01). The *Mecp2^T158A/y^* mice with isotype control antibody treatment made significantly more open-arm entries compared with the wild-type mice at all time points assessed (*p* < 0.05) and more entries than the *Mecp2^T158A/y^* mice with anti-SEMA4D antibody treatment between week 5 and week 9 (*p* < 0.01).

### 2.3. Anti-SEMA4D Therapy Prevents the Onset and Reverses Deficits of Key Locomotor Function

**Figure 2:**
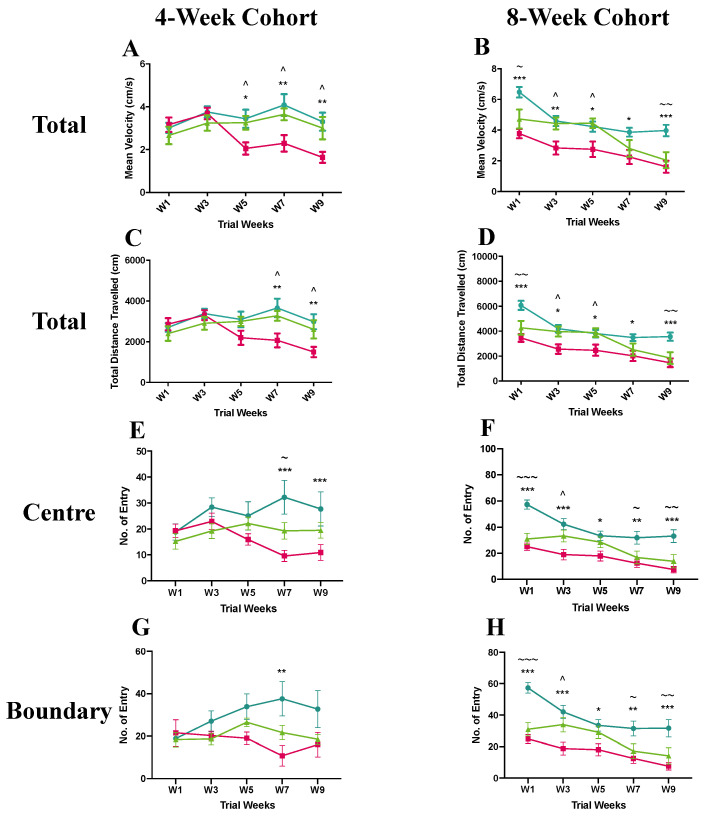
Results of open field test of 4-week (pre-symptomatic) and 8-week (symptomatic) cohorts in the pre-clinical trial. Graphs display analysis of entry in total (**A** and **C**), central (**E**), and boundary (**G**) areas for the 4-week cohort as well as total (**B** and **D**), central (**F**), and boundary (**H**) areas for the 8-week cohort. Data are expressed as mean ± standard error of the mean. 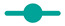 C57BL/6 mice treated with control antibody (WT), 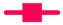 *Mecp2^T158A/y^* mice treated with control antibody (P), 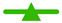 *Mecp2^T158A/y^* mice treated with anti-semaphorin 4D antibody (T). * WT vs. P, ~ WT vs. T, ^ P vs. T. *, ~, and ^ *p* < 0.05; **, ~~, and ^^ *p* < 0.01; ***, ~~~, and ^^^ *p* < 0.001.

To investigate whether the anti-SEMA4D immunotherapy ameliorates locomotor dysfunction, the open field test was utilised with multiple parameters measured in three areas of the open field (total, centre, and boundary). In the pre-symptomatic cohort, the *Mecp2^T158A/y^* mice receiving isotype control antibody treatment showed a significantly lower mean velocity in the total area compared to the wild-type and anti-SEMA4D antibody-treated mice between week 5 and 9 (*p* < 0.05) (Figure 2A). The distance travelled in the total area in the placebo group was also significantly less compared to that of the other two groups in week 7 and week 9 (*p* < 0.05) (Figure 2C). Compared to the wild-type mice, the placebo-treated *Mecp2^T158A/y^* mice made significantly fewer entries into the central area in week 7 and week 9 (*p* < 0.001) and the mice in the anti-SEMA4D treatment group showed a lower number of central area entries than wild-type mice in week 7 (*p* < 0.05) (Figure 2E). The *Mecp2^T158A/y^* mice receiving isotype control antibody demonstrated fewer entries into the boundary area compared with the wild-type mice in week 7 (*p* < 0.01) (Figure 2G).

To determine whether the anti-SEMA4D antibody treatment improves locomotor functions if administered after symptom onset, symptomatic mice receiving anti-SEMA4D treatment or placebo along with the wild-type controls were tested in the open field apparatus. In the total area, the *Mecp2^T158A/y^* mice treated with isotype control antibody showed a significantly lower mean velocity and total distance travelled compared to the wild-type mice throughout the 10-week trial (*p* < 0.05) (Figure 2B,D). The anti-SEMA4D antibody treatment significantly improved the mean velocity and total distance travelled in the *Mecp2^T158A/y^* mice compared to the isotype control antibody in week 3 and 5 (*p* < 0.05) but not week 7 and 9. Similarly, in the central and boundary areas, the isotype antibody-treated *Mecp2^T158A/y^* mice demonstrated significantly fewer entries than wild-type mice throughout the 10-week trial period (*p* < 0.05), and the anti-SEMA4D antibody treatment significantly improved the number of entries into the central and boundary areas in the *Mecp2^T158A/y^* mice compared with the isotype control antibody in week 3 (*p* < 0.05) but not in week 5–9 (Figure 2F,H). Additional measurements for the open field test are supplied in Supplementary Figure S2.

In the pre-symptomatic cohort, effects of anti-SEMA4D antibody were sustained throughout the 10-week treatment period, while in symptomatic mice, activity in the open field test appeared to be reduced after week 5, which coincided with a reduced dose from twice weekly to once weekly of anti-SEMA4D antibody in this cohort. To determine a potential dose-dependent effect, serum antibody levels were assessed at the endpoint. In previous pre-clinical studies, it was determined that target saturation requires at least 5 μg/mL of antibody and corresponds with the minimal effective dose in mice [20,21]. Assuming ~0.1–0.3% penetrance of antibody across the blood–brain barrier [22,23], this would require serum antibody levels of at least ~5 mg/mL. Serum drug levels in the pre-symptomatic cohort (dosed twice weekly) were determined to be 4.96 ± 0.71 mg/mL, in contrast to 2.91 ± 0.55 mg/mL detected following reduction to once weekly dosing in the symptomatic mice. This analysis suggests the possibility of a dose-dependent drug effect in these mice.

### 2.4. Anti-SEMA4D Therapy Prevents the Onset and Reverses Apnoea and Key Respiratory Deficits

**Figure 3:**
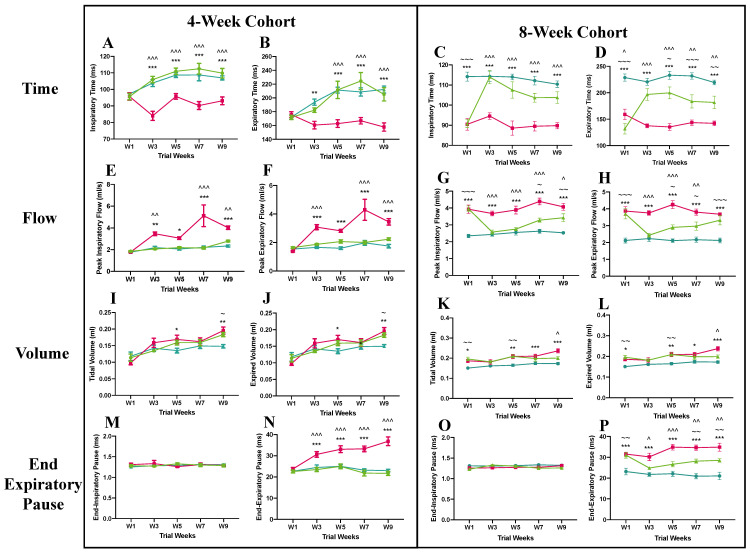
Results of whole-body plethysmography test of 4-week (pre-symptomatic) and 8-week (symptomatic) cohorts in the pre-clinical trial. Graphs display analysis of respiratory time (**A**,**B**), peak respiratory flow (**E**,**F**), maximum volume (**I,J**), and end-respiratory pause (**M**,**N**) for the 4-week cohort as well as respiratory time (**C**,**D**), peak respiratory flow (**G**,**H**), maximum volume (**K**,**L**), and end-respiratory pause (**O**,**P**) for the 8-week cohort. Data are expressed as mean ± standard error of the mean. 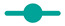 C57BL/6 mice treated with control antibody (WT), 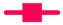 *Mecp2^T158A/y^* mice treated with control antibody (P), 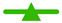 *Mecp2^T158A/y^* mice treated with anti-semaphorin 4D antibody (T). * WT vs. P, ~ WT vs. T, ^ P vs. T. *, ~, and ^ *p* < 0.05; **, ~~, and ^^ *p* < 0.01; ***, ~~~, and ^^^ *p* < 0.001.

In the pre-symptomatic cohort, the *Mecp2^T158A/y^* mice treated with anti-SEMA4D antibody showed a respiratory pattern similar to that of the wild-type mice in all parameters measured, including inspiratory and expiratory time (Figure 3A,B), peak inspiratory and expiratory flow (Figure 3E,F), tidal and expired volume (Figure 3I,J), and end-inspiratory and expiratory pause (Figure 3M,N). In contrast, the placebo-treated *Mecp2^T158A/y^* mice demonstrated an irregular respiratory pattern, with significantly lower inspiratory and expiratory time between week 3 and week 9 (*p* < 0.01) and higher peak inspiratory and expiratory flow from week 3 to week 9 (*p* < 0.05), compared with the wild-type mice. The mice in the placebo group were also found to have higher tidal and expired volume than the wild-type mice in week 5 and week 9 (*p* < 0.05). Interestingly, the end-expiratory pause (EEP), an indicator of apnoea, of the isotype control antibody-treated *Mecp2^T158A/y^* mice progressively increased over the 10-week trial and was significantly higher than that of the wild-type and anti-SEMA4D-treated mice from week 3 to week 9 (*p* < 0.001).

In the symptomatic cohort, the *Mecp2^T158A/y^* mice treated with isotype control antibody demonstrated an irregular pattern of respiration throughout the trial, with reduced inspiratory and expiratory time (*p* < 0.001) (Figure 3C,D) and increased peak inspiratory and expiratory flow (*p* < 0.001) (Figure 3G,H) compared with the wild-type controls. The anti-SEMA4D antibody treatment significantly improved the irregularity of respiratory pattern in the *Mecp2^T158A/y^* mice, with increased inspiratory and expiratory time from week 3 until the end of the trial (*p* < 0.01) and decreased peak inspiratory and expiratory flow between week 3 and week 7 (*p* < 0.05), compared with the isotype control antibody-treated mice. The *Mecp2^T158A/y^* mice in the placebo group showed a higher tidal and expired volume than the wild-type mice in week 1 (*p* < 0.05), week 5 (*p* < 0.01), week 7 (*p* < 0.05), and week 9 (*p* < 0.001) (Figure 3K,L). The tidal and expired volume in the anti-SEMA4D treatment group was significantly lower than that in the placebo group in week 9 (*p* < 0.05). There were no differences in the end-inspiratory pause of any groups throughout the course (Figure 3O). The end-expiratory pause (EEP) of the anti-SEMA4D-treated *Mecp2^T158A/y^* mice was significantly lower than that of the placebo treated *Mecp2^T158A/y^* mice from week 3 (*p* < 0.05) to week 9 (*p* < 0.01) (Figure 3P). Data for additional parameters measured in the whole-body plethysmography test are available in Supplementary Figure S3.

### 2.5. SEMA4D Is Upregulated in Rett Syndrome Neurons and Anti-SEMA4D Therapy Reduces Activation of Receptor-Positive Glial Cells

**Figure 4:**
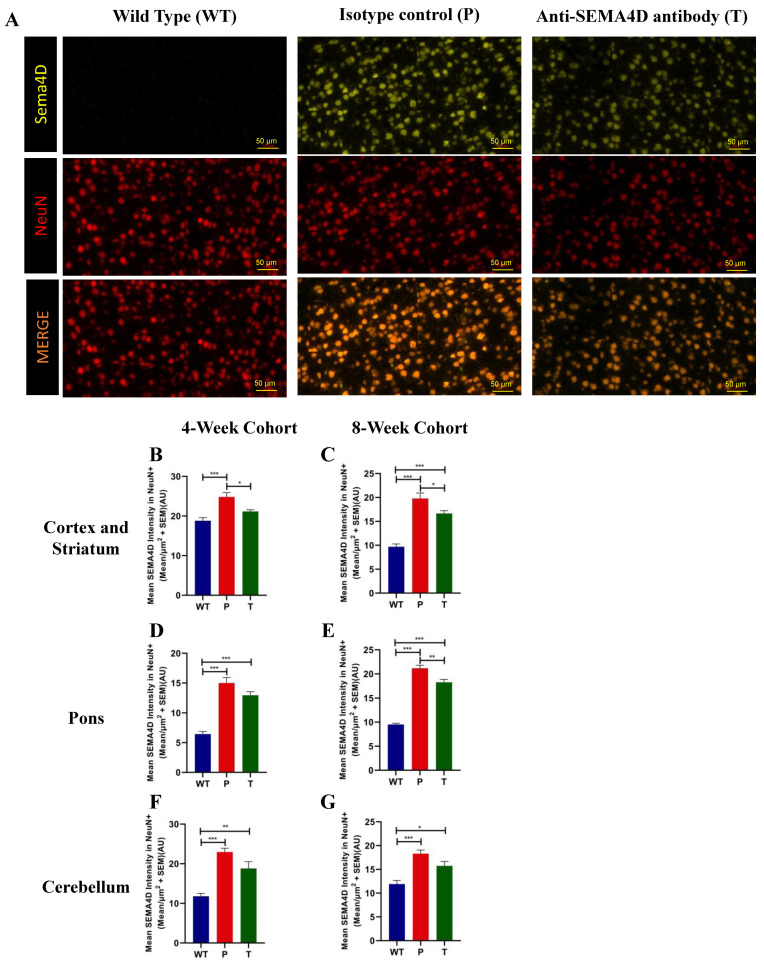
Semaphorin 4D (SEMA4D) and neuronal (NeuN) expression in brains of C57BL/6 (WT) and *Mecp2^T158A/y^* mice. (**A**) Representative images of immunohistochemistry SEMA4D and NeuN staining in the somatomotor cortex of age-matched mice from the 8-week cohort, C57BL/6 (WT), and *Mecp2^T158A/y^* treated with isotype control (P) or anti-SEMA4D antibody (T). (**B**–**G**) Mean staining intensity of SEMA4D in NeuN^+^ neuronal cells and was measured in the cortex and striatum, the pons, and the cerebellum. For immunohistochemistry, three consecutive mouse brain sagittal serial sections (10 µm), 25 µm apart, from each mouse, were evaluated. Three sections/mouse were averaged for each mouse, and group means are an average of 6 mice/group. Data are expressed as group mean ± standard error of the mean. * *p* < 0.05; ** *p* < 0.01; *** *p* < 0.001.

In the male *Mecp2^T158A/y^* mice, we observed a dramatic increase in SEMA4D colocalising with neuronal marker NeuN, suggestive of the neuronal origin of SEMA4D in the diseased brain (Figure 4A). In contrast, expression of SEMA4D in adult brains of wild-type mice was low/undetectable. Upregulation of SEMA4D was also observed in brains of female *Mecp2^T158A/+^* mice (data not shown). The mean SEMA4D intensity in NeuN+ neuronal cells was significantly elevated in the cortex and striatum (Figure 4B,C), the pons (Figure 4D,E), and the cerebellum (Figure 4F,G) of the *Mecp2^T158A/y^* mice treated with isotype control antibody, compared with the WT mice in both pre-symptomatic and symptomatic cohorts (*p* < 0.001). In *Mecp2^T158A/y^* mice treated with anti-SEMA4D antibody, the mean SEMA4D intensity in NeuN+ neuronal cells was slightly reduced relative to *Mecp2^T158A/y^* mice treated with isotype control in the cortex and striatum in both pre-symptomatic and symptomatic cohorts (*p* < 0.05), as well as in the pons in the symptomatic cohort (*p* < 0.01). It appears that antibody binding promotes only limited internalisation of SEMA4D in neurons, as SEMA4D levels in treated mice, however, remained significantly elevated relative to wild-type mice [20].

**Figure 5:**
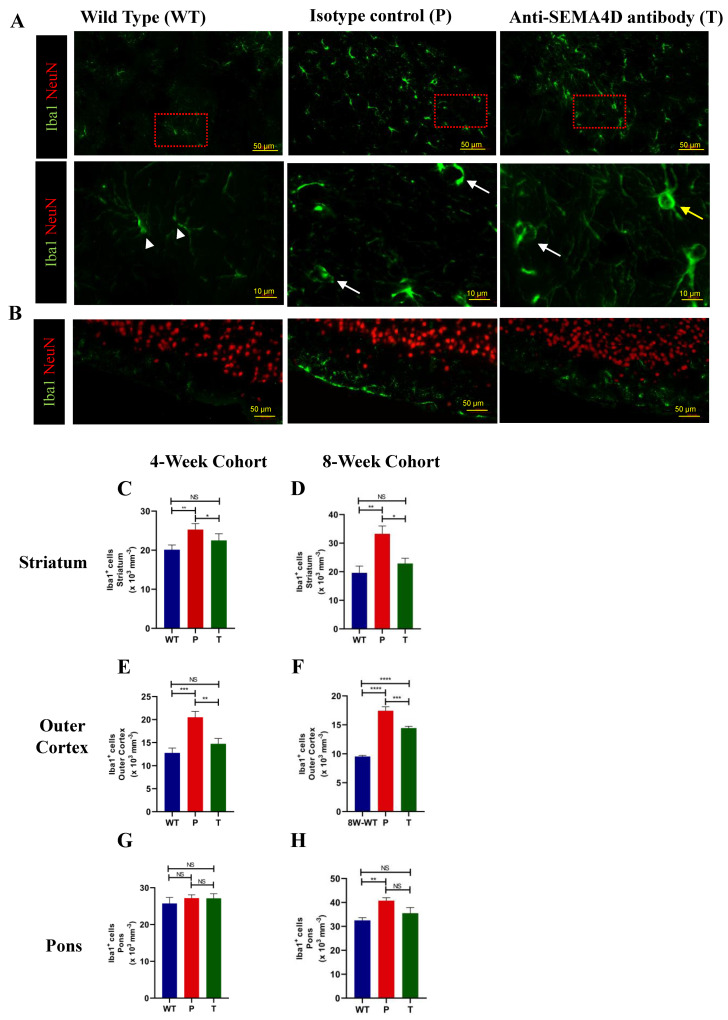
Ionized calcium-binding adaptor protein-1 (Iba1)^+^ cells in brains of C57BL/6 (WT) and *Mecp2^T158A/y^* mice. (**A**) Representative images of immunohistochemistry Iba1 staining in the striatum of age-matched mice from the 8-week cohort, C57BL/6 (WT), and *Mecp2^T158A/y^* treated with isotype control (P) or anti-SEMA4D antibody (T). Lower panel represents high-magnification images of Iba1+ cells with various morphologies; arrowhead = ramified, white arrow = amoeboid, yellow arrow = amoeboid with stout. (**B**) Representative images of immunohistochemistry Iba1 staining in the outer cortex including pia mater. (**C**–**H**) Quantification of Iba1+ cell density in the striatum, outer cortex and pons. For immunohistochemistry, three consecutive mouse brain sagittal serial sections (10 µm), 25 µm apart, from each mouse, were evaluated to determine the number of Iba1+ cells. Three sections/mouse were averaged for each mouse, and group means are an average of 6 mice/group. Data are expressed as group mean ± standard error of the mean. * *p* < 0.05; ** *p* < 0.01; *** *p* < 0.001, **** *p* < 0.0001.

To assess effects on glial cells that express receptors for SEMA4D, microglia were stained using antibodies targeting Iba1 (Figure 5). Microglia in wild-type mice display healthy resting ramified morphology while microglia from *Mecp2^T158A/y^* mice appear to increase in number and exhibit an amoeboid shape indicative of activation, as shown in representative high-magnification images from the striatum (second row, Figure 5A). Treatment with anti-SEMA4D reduced the number of Iba1+ cells in the striatum and appeared to alter the morphology in the *Mecp2^T158A/y^* mice to a mixture of ramified and amoeboid microglia. Accumulation of Iba1+ cells was observed in the outer cortex of *Mecp2^T158A/y^* mice, which was partially inhibited in *Mecp2^T158A/y^* mice treated with anti-SEMA4D antibody (Figure 5B). A significant increase in the number of Iba1+ cells in the *Mecp2^T158A/y^* mice was observed in the striatum (Figure 5C,D), outer cortex (Figure 5E,F), and pons (Figure 5G,H), compared with the WT mice in both pre-symptomatic and symptomatic cohorts (*p* < 0.01). Antibody treatment significantly reduced levels of Iba1+ cells in the striatum (*p* < 0.01) and outer layer of the cortex (*p* < 0.05), and a trend of reduced Iba1+ cells following treatment was observed in the pons (Figure 5G,H). No difference was observed between Rett syndrome and WT in other cortical regions or the cerebellum (data not shown).

**Figure 6:**
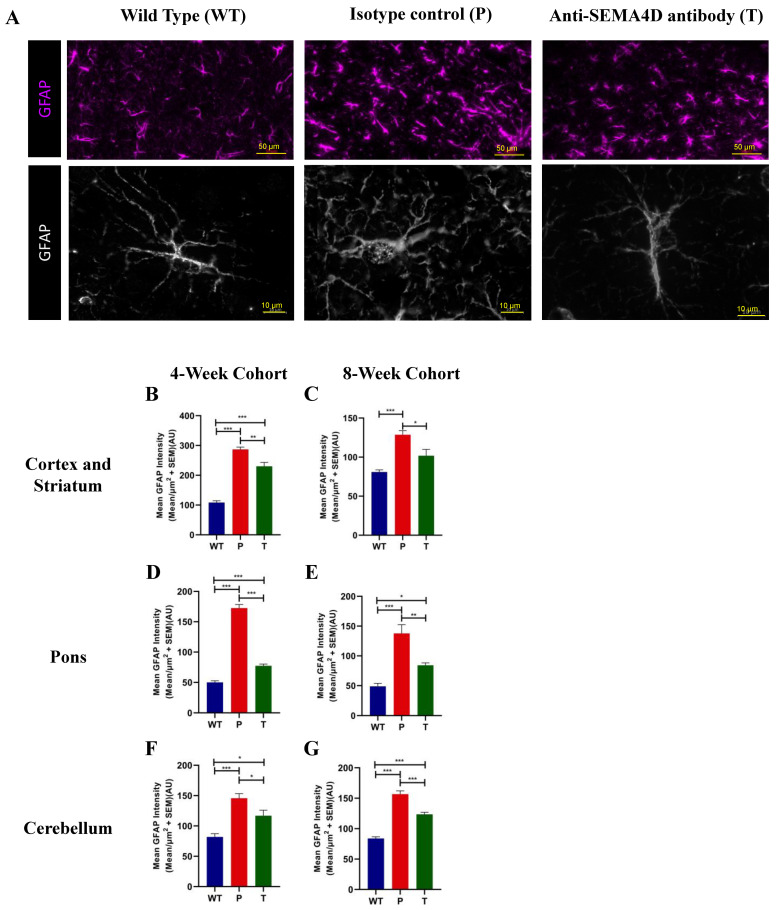
Glial fibrillary acidic protein (GFAP) expression in brains of C57BL/6 (WT) and *Mecp2^T158A/y^* mice. (**A**) Representative images of immunohistochemistry GFAP staining in the somatomotor cortex of age-matched mice from the 8-week cohort, C57BL/6 (WT), and *Mecp2^T158A/y^* mice treated with isotype control (P) or anti-SEMA4D antibody (T); lower panel shows high magnification for finer resolution of astrocyte morphology. (**B**–**G**) Mean staining intensity of GFAP was measured in the cortex and striatum, the pons, and the cerebellum. For immunohistochemistry, three consecutive mouse brain sagittal serial sections (10 µm), 25 µm apart, from each mouse, were evaluated. Three sections/mouse were averaged for each mouse, and group means are an average of 6 mice/group. Data are expressed as group mean ± standard error of the mean. * *p* < 0.05; ** *p* < 0.01; *** *p* < 0.001.

Astrocytes play an important role in cross talk with neurons and microglia to propagate neuroinflammation that can exacerbate degenerative pathology in the setting of chronic stimulation. Astrocytes were stained for GFAP, a predominantly astrocytic marker which is upregulated in reactive astrocytes. The mean GFAP intensity was significantly increased in *Mecp2^T158A/y^* mice (Figure 6A), predominantly in the cortex and striatum (Figure 6B,C), the pons (Figure 6D,E), and the cerebellum (Figure 6F,G) compared with the WT mice in both pre-symptomatic and symptomatic cohorts (*p* < 0.001). Morphometric changes, as evidenced by increased soma size and reduced hypertrophy of astrocyte processes and significant reduction in GFAP, are indicative of astrocyte activation [6]. Treatment with anti-SEMA4D antibody significantly decreased the mean GFAP intensity compared to placebo-treated *Mecp2^T158A/y^* mice in the cortex and striatum (*p* < 0.05), the pons (*p* < 0.01), and the cerebellum (*p* < 0.05) in both cohorts.

### 2.6. Anti-SEMA4D Mitigates the Abnormal Cytoskeletal Structure in Astrocytes Isolated from Mecp2^T158A/y^ Mutant Mice

**Figure 7:**
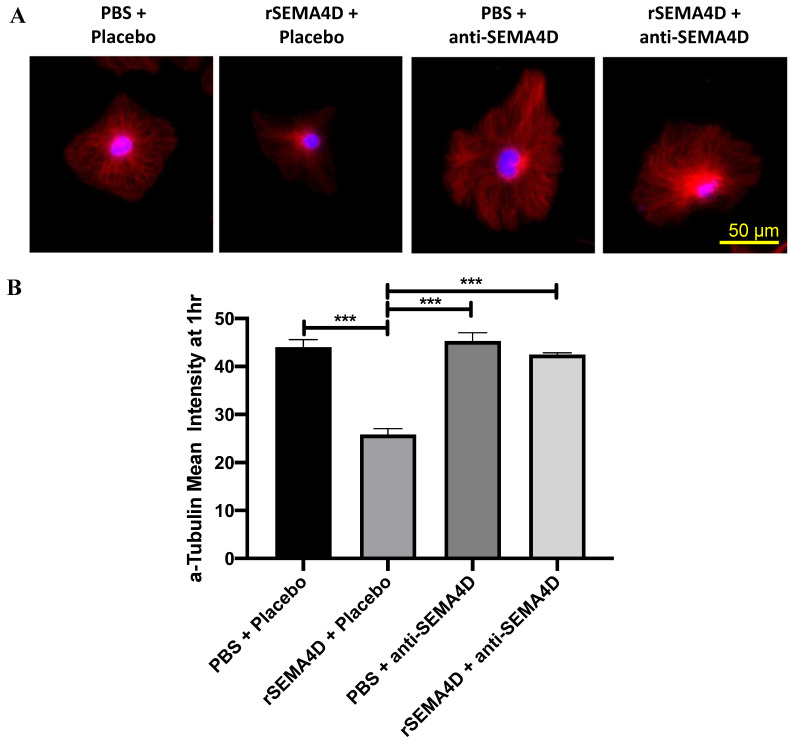
Effects of anti-semaphorin 4D (SEMA4D) antibody on the cytoskeleton. (**A**) Immunofluorescence shows α-tubulin (red) and nuclei (blue) in *Mecp2^T158A/y^* mixed glial culture exposed to recombinant semaphorin 4D (rSEMA4D) or phosphate-buffered saline (PBS) and treated with anti-SEMA4D or isotype control (placebo) antibody for 1 h. (**B**) Mean intensity of α-tubulin staining in GFAP+ (not shown) astrocytes was quantified and expressed as mean ± standard error of the mean. *** *p* < 0.001.

To investigate the mechanism of action of anti-SEMA4D antibody on glial cells, mixed glial cells were treated with rSEMA4D alone or together with anti-SEMA4D blocking antibody. The cytoskeletal changes in response to rSEMA4D and anti-SEMA4D antibody were assessed by microtubule expression (α-tubulin immunofluorescence) in GFAP-positive astrocytes. GFAP-positive astrocytes express PLXNB1 receptor for SEMA4D. Representative images of immunofluorescent staining of α-tubulin (red) with nuclear counterstain of Hoechst (blue) in GFAP-positive astrocytes (staining not shown) are presented in Figure 7A. The levels of α-tubulin immunofluorescence intensity were reduced when incubated with rSEMA4D (*p* < 0.001 compared with the PBS groups), which was rescued by anti-SEMA4D antibody treatment (*p* < 0.001 PBS + anti-SEMA4D versus. rSEMA4D + anti-SEMA4D; *p* < 0.001, rSEMA4D + placebo versus. rSEMA4D + anti-SEMA4D antibody) (Figure 7B).

## 3. Discussion

Here, we have demonstrated the therapeutic efficacy of anti-SEMA4D antibody treatment in alleviating phenotypic symptoms and functional deficits in a transgenic mouse model of Rett syndrome. We show that anti-SEMA4D antibody therapy not only prevents, but reverses, many Rett syndrome-specific phenotypes, including coordination, cognition, locomotion, and respiratory deficits in both pre-symptomatic and symptomatic mice. The beneficial effects of anti-SEMA4D antibody therapy observed in the pre-clinical trial may be a result of antibody blockade of SEMA4D-mediated Plexin B1 signal transduction in glial cells, as previously suggested [13].

Rett syndrome mice share several symptoms with Rett syndrome patients, such as stereotypic movements and body tremor [24,25]. Hind limb clasping in Rett syndrome mice is a postural response that resembles the characteristic hand wringing stereotypies present in Rett syndrome patients [24]. Many adult Rett syndrome patients present body tremors, which appear later in the evolution of the disease [26]. In the current study, the *Mecp2^T158A/y^* mice receiving isotype control antibody started developing classic Rett syndrome-specific phenotypes at 5 weeks of age which persisted to the end of trial, in line with previous studies [27,28,29]. Our results indicate that anti-SEMA4D antibody therapy improves the characteristic posturing stereotypes and reduced the body tremor regardless of whether it was administered during the pre-symptomatic or symptomatic stage of the disorder.

No significant differences were observed in the survival of *Mecp2^T158A/y^* treated and untreated mice cohorts. Rett syndrome predominantly affects girls where individuals have the potential for prolonged survival with approximately 60% surviving to early middle age while Rett syndrome is rare in males and they usually succumb to death before birth or in early infancy owing to only having one X chromosome [30]. In this study, we utilised the male *Mecp2^T158A/y^* model due to its robust phenotype which includes a significantly shorter survival rate which is not observed in females with Rett syndrome. Rett syndrome patients experience deterioration of motor coordination skills that impact sensation, movement, and communication in early childhood. In this study, we demonstrate the prevention and reversal of motor coordination and cognitive deficits (as observed by the rotarod test and the elevated plus maze test) in anti-SEMA4D-treated *Mecp2^T158A/y^* mice, whereas *Mecp2^T158A/y^* mice treated with control antibody exhibited deficits as early as 6 weeks of age which progressively worsened throughout the trial. Surprisingly, the treated mice in the symptomatic cohort stayed longer on the rotarod than the WT mice in week 7, suggesting a potential improvement in motor learning ability. This may be further assessed by conducting the accelerating rotarod test in one session per day for 5 consecutive days, then comparing the performance on the fifth day to that on the first [31].

The locomotor deficits (open field test) observed in the isotype control antibody-treated *Mecp2^T158A/y^* mice in both the pre-symptomatic and symptomatic cohorts were consistent with the locomotor impairments reported in previous studies of the *Mecp2^T158A^* mouse model [27,28]. The locomotor deficits in Rett syndrome have been postulated to be caused by astrocytic dysfunctions (oxidative phosphorylation, potassium and neurotransmitter homeostasis, and hyperreactivity) [32,33,34,35] and microglia-induced inflammatory responses [4,33,36]. Anti-SEMA4D immunotherapy demonstrated a measurable improvement of locomotor function in both pre-symptomatic and symptomatic mice during the trial. In the area-specific analysis of the symptomatic cohort, significant improvements were found in the anti-SEMA4D treatment group between week 3 and 5 but not detected during the rest of trial, corresponding to the reduction in the antibody dose from twice weekly to once weekly starting at week 5. These results suggest that the improvements observed in the locomotor function may be dose dependent. Further dosing comparison and wash-out studies will be required to further optimise the dosing regimen. It is also possible that differences in activity observed between the pre-symptomatic and symptomatic cohorts resulted from the stage of disease at the time of treatment initiation.

Rett syndrome patients suffer from respiratory symptoms including hyperventilation, apnoea, forced deep breathing, apneustic breathing, and severely arrhythmic breathing, profoundly affecting quality of life [37]. These respiratory symptoms in Rett syndrome are believed to be a result of aberrant expression of neurotransmitters and neuromodulators in astrocytes [37]. Recent studies show that arrhythmic breathing is caused by impaired carbon dioxide (CO_2_) chemosensitivity of astrocytes [38,39]. In the current study, data generated from the whole-body plethysmography test indicate the irregular pattern of respiratory time, flow, and volume (both inspiratory and expiratory), as well as the end-expiratory pause (apnoea) observed in the *Mecp2^T158A/y^* mice treated with isotype control antibody. Our results indicate that anti-SEMA4D treatment prevents and improves arrhythmic breathing in pre-symptomatic and symptomatic mice. Interestingly, the respiratory improvements in the symptomatic cohort follow the same dose-dependent pattern found in the area-specific analysis of the open field test.

The upregulation of SEMA4D/Plexin B1 signalling and the importance of neuroglia interactions driving the pathology of Rett syndrome provide a mechanistic rationale for the amelioration of Rett syndrome-specific symptoms and the improvement of behavioural functions in anti-SEMA4D antibody-treated *Mecp2^T158A/y^* mice. SEMA4D was shown to be upregulated in neurons of *Mecp2^T158A/y^* mice, perhaps in response to stress induced by aberrant Mecp2 expression. The interaction of SEMA4D molecules and Plexin B1 receptors on glial cells has been reported to activate nuclear factor kappa-light-chain-enhancer of activated B cells (NF-κB), Ras homolog family member A (RhoA), and Ras related protein (R-Ras) pathways, leading to cytoskeletal restructuring in astrocytes and inflammatory responses in microglia via the NF-κB, RhoA, and R-Ras signalling pathways [14,40]. As shown here, an increased number of GFAP+ astrocytes and Iba1+ cells, together with characteristic morphologic changes, suggests an increase in reactive gliosis in the *Mecp2^T158A/y^* mice, which is reduced following treatment with SEMA4D blocking antibody. This suggests a model whereby *MECP2* mutations induce neuronal stress to trigger neuronal expression of SEMA4D, which then binds to cognate Plexin B1 receptors to trigger astrocyte reactivity and microglial activation. By blocking SEMA4D–Plexin B1 binding, the anti-SEMA4D antibody may alleviate the cellular and inflammatory responses in glial cells, manifested as symptomatic relief and functional improvement in the *Mecp2^T158A/y^* mice.

We found marked activation of glial cells in the brain, including the striatum and pons regions, which registered a significant increase in cells expressing GFAP and Iba1 in *Mecp2^T158A/y^* mice treated with placebo compared to wild-type littermate controls and significant lowering of activated astrocytes and microglia with SEMA4D antibody treatment. These observations are highly pertinent to Rett neuropathology, as *Mecp2* mutations are associated with impairments in locomotion and motor skill learning, which directly relates to the functionality of striatal motor control. Similarly, *Mecp2* mutant mice show respiratory problems, including dysrhythmia and apnea, which are attributed to the malfunctioning/hyperexcitability of the pons [41,42]. Interestingly, SEMA4D levels were somewhat reduced in neurons of *Mecp2^T158A/y^* mice following treatment. One possibility is that antibody treatment reduced stress and restored health of neurons which reduced SEMA4D expression. Another possibility is that antibody binding to SEMA4D on neurons resulted in internalisation of antigen–antibody complexes, as reported previously [43], and they were possibly targeted for degradation. We cannot rule out the possibility that peripheral effects of SEMA/Plexin signalling may also play a role, as Plexin B1 and Plexin B2 receptors are also expressed by myeloid cells in the periphery and SEMA4D is expressed on lymphocytes. MeCP2 deficiencies have been reported to alter immune responses and interactions between lymphocytes and myeloid cells [4]. Iba1+ cells at the cortical border may represent non-microglial myeloid cells [41], however, ramified Iba1+ cells deep within the striatum are likely to be microglia whose reduced process complexity is indicative of activation in the *Mecp2^T158A/y^* mice [4]. Detailed mechanistic studies are required to characterise and further investigate how the *Mecp2* mutations affect Plexin B1 expression in glial cells, and effects of SEMA4D on neuronal function and phenotype, as well as the impact on peripheral immune responses that can also influence neuropathology of Rett syndrome.

The behavioural impairment observed in Rett syndrome is believed to be heavily affected by *Mecp2* deficiency or malfunction in microglia and astrocytes in various regions of the brain [31,42,44,45,46]. By blocking SEMA4D–Plexin B1 binding, the anti-SEMA4D antibody not only prevents, but appears to reverse, the phenotypes and impairment as early as two weeks after treatment in the current study. The rapid response to the anti-SEMA4D antibody therapy in the symptomatic mice suggests anti-SEMA4D immunotherapy may have potential to treat the Rett syndrome girls who are usually diagnosed after symptom onset. In this study, we demonstrated that anti-SEMA4D immunotherapy not only reverses Rett-like symptoms but also prevents them, however, the prevention of Rett syndrome neurological signs and symptoms remains challenging as diagnosis is mostly achieved after these neurological symptoms are evident. Therefore, anti-SEMA4D therapy remains a potential treatment for individuals diagnosed with Rett syndrome until such time that early intervention strategies such as pre-natal testing or early genetic testing are implemented in healthcare systems. Furthermore, anti-SEMA4D immunotherapy has been found to be well tolerated in several clinical trials (NCT01764737, NCT03425461, NCT03373188, NCT03690986, NCT03320330, and NCT03268057), including application in paediatrics (NCT03320330), an important first step in bench-to-bed translation.

To our knowledge, the current study is the first to report the beneficial effects of anti-SEMA4D antibody in preventing and improving the symptoms and deficits in a transgenic mouse model of Rett syndrome. The pre-clinical trial results suggest that anti-SEMA4D immunotherapy is a potentially promising therapeutic strategy to improve phenotype, coordination, cognition, locomotion, and respiration in Rett syndrome.

## 4. Materials and Methods

### 4.1. Animal Model

All experimental procedures were carried out in strict accordance with the Australian Code of Practice for the Care and Use of Animals for Scientific Purposes, National Health and Medical Research Council, and approved at the Children’s Medical Research Institute and the Children’s Hospital by Westmead Joint Animal Ethics Committee (Approval Number K372/2019).

These studies used wild-type (C57BL/6) and the *Mecp2^T158A^* knock-in Rett syndrome mouse model harbouring a p.Thr158Ala variation resulting in a reduction in Mecp2 binding to methylated DNA [27]. The threonine 158 (T158) residue is located at the C-terminus of the methyl-CpG binding domain (MBD) of MeCP2 and represents one of the most common mutations observed in RTT, with the p.Thr158Met mutation being one of 8 hotspot mutations and approximately 10% of all RTT cases carrying this mutation [47]. Although p.Thr158Ala mutations occur at a lower frequency than p.Thr158AMet in RTT patients, the mechanism through which both mutations impair MeCP2 function is believed to be similar as patients carrying the p.Thr158Ala or p.Thr158AMet mutations are phenotypically similar [47,48,49].

*Mecp2^T158A^* mice recapitulate many phenotypic features of Rett syndrome patients, including hypoactivity, motor dysfunction, neurodevelopmental regression, abnormal anxiety levels, and learning and memory deficits. Male hemizygous mice (*Mecp2^T158A/y^*) usually start showing symptoms at 4–6 weeks old, while female heterozygous mice (*Mecp2^T158A/+^*) only show symptoms after 17 weeks. *Mecp2^T158A/y^* mice show characteristic Rett-like phenotypes, including hindlimb clasping, dysregulated weight, and seizure activity. In addition, they spend significantly less time on the rotarod compared to WT littermates, significantly less time in the closed arm and significantly more time in the open arm of the elevated plus maze compared to their WT littermates, with a significant reduction in locomotor activity and distance travelled.

### 4.2. Antibody Treatment

Hemizygous *Mecp2^T158A/y^* and wild-type C57BL/6 male mice were treated with either anti-SEMA4D (Mab 67) or mouse immunoglobulin G1 (IgG1) isotype control monoclonal antibody at 4 weeks old (when the *Mecp2^T158A/y^* mice were pre-symptomatic) or 8 weeks old (when the *Mecp2^T158A/y^* mice were symptomatic) (Table 1). In the 4-week cohort (pre-symptomatic), all mice were dosed twice weekly with 50 mg/kg anti-SEMA4D (treatment) or an isotype control (placebo) antibody diluted in phosphate-buffered saline (PBS) by intraperitoneal injection for 10 weeks. In the 8-week cohort (symptomatic), all mice received intraperitoneal injections of 50 mg/kg anti-SEMA4D or isotype control antibody twice weekly in the first 4 weeks and then once weekly between week 5 and 10 of the trial.

### 4.3. Behavioural Assessments

Phenotypic scoring was performed on a weekly basis for the severity of Rett syndrome-specific symptoms, including tremor and hindlimb clasping, according to the criteria in Table 2. Every two weeks, physiological, locomotor, and cognitive outcome measures were assessed using syndrome-specific symptoms and the elevated plus maze, accelerating rotarod, open field, and whole-body plethysmography tests. These behavioural tests were conducted over 3–4 days to reduce anxiety and fatigue in the mice.

Elevated Plus Maze. Each mouse was placed in the cross-section of the elevated plus maze at 75 cm height for 5 min as previously published [50]. The time spent in the closed and open arms and the number of entries into closed and open arms were recorded.

Accelerating Rotarod. Mice were placed on an accelerating rotarod apparatus for 3 trials with at least 15 min of rest between trials. Each trial lasted for a maximum 3 min, during which the rod accelerated 5 rpm every 15 s from 5 to 60 rpm. The latency to fall from the rod was recorded for each trial.

Open Field. At the beginning of each trial, the mouse was placed at the centre of the open field apparatus (LE802S, Panlab Harvard Apparatus, Barcelona, Spain) for 15 min. The subject’s behaviour was tracked and recorded by ActiTrack software (v2.7 Panlab Harvard Apparatus, Barcelona, Spain) for the duration of each trial.

Whole Body Plethysmography. Each mouse was placed individually in a chamber of the whole-body plethysmography apparatus (vent 4, SCIREQ, Montreal, QC, Canada) in a dark room for 3 h without disturbance. The breathing pattern of each animal was recorded and analysed by EMKA iox software (v2.10.5, SCIREQ, Montreal, QC, Canada).

### 4.4. Immunohistochemistry

Brains were coronally dissected and formalin fixed and paraffin embedded (FFPE). Left brain hemispheres were immersion fixed in formalin followed with paraffin embedding for immunohistochemistry. Three consecutive mouse brain sagittal serial sections (10 µm), 25 µm apart, from 6 mice per cohort, were evaluated. Fluorescent immunohistochemistry staining was performed for mouse semaphorin 4D (PA5-47711, Invitrogen, Waltham, MA, USA), NeuN (ab177487, Abcam, Cambridge, UK), Iba1 (ab178846, Abcam), and GFAP (ab7260, Abcam), in accordance with manufacturer-recommended concentrations combining host-dependent secondary Alexa Fluor antibodies. For the same host species primary antibodies, monovalent Fab fragment antibodies (111007003, 711007003, Jackson ImmunoResearch, West Grove, PA, USA) were used for blocking along with sequential staining. Imaging was performed on an AxioObserver7 Automated Inverted Microscope with Plan-Apochromat 40×/0.95 objective and Axiocam 702 Monochrome Camera.

For analysis of immunofluorescence intensity and cell numbers, whole tissue sections were mounted on a slide, with three consecutive slices per sample, and images of stained samples were analysed with Fiji/ImageJ software (National Institutes of Health, Bethesda, MD, USA, http://imagej.nih.gov/ij/, version 1.53C, accessed on 30 August 2021). Cell densities were calculated as the mean from three consecutive sections from a sum of the numbers for each image divided by the area of the region selected. For Iba1, Z-stack images from three consecutive mouse brain sagittal serial sections (10–25 µm apart) were used for cell density calculations.

### 4.5. Pharmacokinetics Analysis

Mouse serum samples were pooled into groups of 3–4 mice/pool, with 3 pools per experimental group for analysis via enzyme-linked immunosorbent assay (ELISA). Serum pools were diluted 1:900 in 1× PBS buffer, 0.5% bovine serum albumin (AP-4510-01, SeraCare, Milford, MA, USA), 0.02% Tween-20 (EC-607, National Diagnostics, Atlanta, GA, USA). Plates were coated overnight with rSEMA4D (Vaccinex, Inc., Rochester, NY, USA) at 1.7 µg/mL in PBS, blocked for 1 h, and incubated with titrated serum samples for 2 h. Drug was detected using goat anti-mouse IgG1-HRP (1:10,000, 1070-05, SouthernBiotech, Birmingham, AL, USA) and developed with TMB substrate (TMBW-0100-01, Surmodics, Eden Praire, MN, USA). Plates were read at 450 nm using a BioTek™ Powerwave™ 340 plate reader, and drug concentrations were calculated based on the standard curve.

### 4.6. Cell Culture

Mixed glial cell cultures were prepared from post-natal day 3 (P3) male mice. Brains were removed and placed in modified Eagle’s medium (MEM, 11090081, Thermo Fisher, Waltham, MA, USA) containing 25 mM N-2-hydroxyethylpiperazine-N-2-ethane sulfonic acid (HEPES, 15630080, Thermo Fisher, Waltham, MA, USA). After removal of the meninges, cerebral cortices were chopped and incubated with 1 mL MEM-HEPES solution containing 1 mg papain (LS003126, Worthington, OH, USA), 240 μg L-cysteine (C7352, Sigma-Aldrich, St. Louis, MO, USA), and 1140 units of DNase I type IV (D5025, Sigma-Aldrich, St. Louis, MO, USA) at 37 °C for 1 h. Supernatant was removed, and the tissue was washed in 1 mL of medium consisting of high-glucose Dulbecco’s modified Eagle’s medium (DMEM) with L-glutamine (11995065, Thermo Fisher, Waltham, MA, USA), 10% (*v*/*v*) foetal bovine serum (10099141, Thermo Fisher, Waltham, MA, USA), and 100 units/mL penicillin–streptomycin (15140122, Thermo Fisher, Waltham, MA, USA). The brain tissue was then triturated through a 1 mL pipette tip until a homogeneous suspension was obtained, pelleted by centrifugation at 170× *g* for 5 min and resuspended in DF medium. The cell suspension was then added to poly-D-lysine (5 μg/mL, P7405, Sigma-Aldrich, St. Louis, MO, USA)-coated 75 cm^2^ cell culture flasks (T75) containing DF medium. Cells were incubated at 37 °C in an atmosphere of 5% CO_2_, with medium being changed on the following day and then every 3 days. Once confluent, cells were dissociated by TrypLE Express Enzyme (12604021, Thermo Fisher, Waltham, MA, USA) and split at a ratio of 1:6 in poly-D-lysine-coated T75 cell culture flasks.

### 4.7. Immunofluorescence Assays

Mixed mouse glial cells from *Mecp2^T158A/y^* and wild-type male mice were seeded into 48-well plates at 4500 cells per well and incubated at 37 °C with 5% CO_2_ for 48 h. Cultures were exposed to recombinant SEMA4D (Vaccinex, Rochester, NY, USA) or PBS and treated with anti-SEMA4D or isotype control antibody for 1 h. Cells were fixed with 4% paraformaldehyde in PBS for 20 min, permeabilised for 20 min with 1% Triton X-100 (X100, Sigma-Aldrich, St. Louis, MO, USA) in PBS and blocked for 1 h in blocking solution containing 0.5% bovine serum albumin (05470 Sigma-Aldrich, St. Louis, MO, USA) and 0.01% Tween 20 (P1379, Sigma-Aldrich, St. Louis, MO, USA) in PBS. Cells were incubated with primary anti-α-tubulin antibody (1:2000, PA1-38814, Thermo Fisher, Waltham, MA, USA), anti-glial fibrillary acidic protein (GFAP, 1:1000, ab53554, Abcam, Cambridge, UK), and anti-Plexin B1 antibody (1:200, ab90087, Abcam, Cambridge, UK) overnight at 4 °C, followed by appropriate species-specific secondary antibodies (Alexa Fluor 488, 555, or 647, 1:5000, Thermo Fisher, Waltham, MA, USA) for 2 h at room temperature in combination with Hoechst nuclear stain (1:50,000, 62249, Thermo Fisher, Waltham, MA, USA) and washed in PBS for 3 × 5 min. Microscopic images were taken by an inverted microscope (DMi8, Leica, Wetzlar, Germany) and relative intensities per cell were determined using ImageJ software (v1.8.0_172, National Institute of Health, Bethesda, MD, USA).

### 4.8. Statistical Analysis

Analysis of all behavioural and in vitro data was performed using Prism 7 (version 7.02, GraphPad, San Diego, CA, USA). A two-tailed, unpaired Student’s *t*-test was used to determine differences between 2 groups. One-way analyses of variance with Bonferroni post hoc tests were used to determine significant differences for more than 2 groups. Two-way analyses of variance with repeated measures of time followed by Bonferroni post hoc tests were used to determine significant differences in all behavioural assessments. A Kaplan–Meier estimator was used to compare the differences between animal survival of any treatment groups during the pre-clinical trial. Differences were considered statistically significant at *p* < 0.05. Analysis of immunohistochemistry data was performed by IBM SPSS software (version 1.0.0.1327). Group differences among treatment conditions were assessed through one-way ANOVA. All error bars in the figures represent the standard error of the mean (SEM). * *p* < 0.05; ** *p* < 0.01; *** *p* < 0.001; **** *p* < 0.0001.

## 5. Patents

A patent application for the USE OF SEMAPHORIN-4D BINDING MOLECULES FOR THE TREATMENT OF RETT SYNDROME has been lodged (inventors: Y.M., W.A.G., E.E.E., M.Z.).

## Figures and Tables

**Table 1:** Experimental plan for the pre-clinical trial.

Mouse Group	Age at Start of Treatment	Treatment Type	Treatment Duration	N =
Wild type	4 weeks old	Isotype control	10 weeks	10
Pre-symptomatic *Mecp2^T158A/y^*	4 weeks old	Isotype control	10 weeks	11
Pre-symptomatic *Mecp2^T158A/y^*	4 weeks old	Anti-SEMA4D	10 weeks	11
Wild type	8 weeks old	Isotype control	10 weeks	10
Symptomatic *Mecp2^T158A/y^*	8 weeks old	Isotype control	10 weeks	12
Symptomatic *Mecp2^T158A/y^*	8 weeks old	Anti-SEMA4D	10 weeks	10

**Table 2:** Scoring criteria for Rett syndrome-specific symptoms in *Mecp2^T158A^* mice.

Score	Tremor	Hindlimb Clasping
0	Not visible or sensible when held in hands	Hindlimbs consistently splayed outward, away from the abdomen
1	Not visible but sensible when held in hands	One hindlimb retracted or both hindlimbs partially retracted toward the abdomen
2	Visible and sensible when held in hands	Hindlimbs entirely retracted and touching the abdomen

## Supplementary Materials

The following are available online at https://www.mdpi.com/article/10.3390/ijms22179465/s1, Figure S1: Survival curves (A,B) and body weight graphs (C,D) of 4-week (pre-symptomatic) and 8-week (symptomatic) cohorts in the pre-clinical trial, Figure S2: Additional results of open field test of 4-week (pre-symptomatic) and 8-week (symptomatic) cohorts in the pre-clinical trial, Figure S3: Additional results of whole-body plethysmography test of 4-week (pre-symptomatic) and 8-week (symptomatic) cohorts in the pre-clinical trial.
