# Supplementary material for: Anti-Semaphorin 4D Rescues Motor, Cognitive, and Respiratory Phenotypes in a Rett Syndrome Mouse Model
Source: Int J Mol Sci. 2021 Aug 31;22(17):9465. doi: 10.3390/ijms22179465 (PMC8431088; doi:10.3390/ijms22179465)
Supplement: Supplementary file 1 [file ijms-22-09465-s001.zip › ijms-1331034-supplementary.pdf]

Supplement data for:

Anti-Semaphorin 4D rescues motor, cognitive and respiratory phenotypes in a Rett syndrome mouse model

Yilin Mao[1,2], Elizabeth E. Evans[5], Vikas Mishra[5], Leslie Balch[5], Allison Eberhardt[5], Maurice Zauderer[5†], Wendy A. Gold[1,2,3,4*†]

[†]Denotes co-last authors.

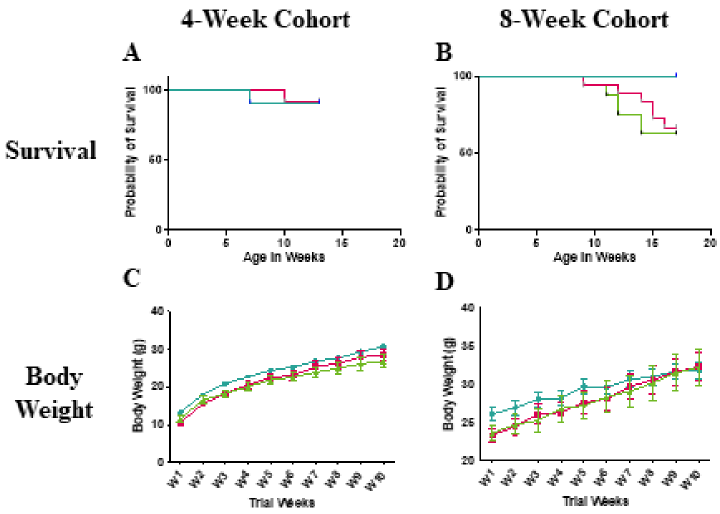

Supplementary Figure S1. Survival curves (**A–B**) and body weight graphs (**C–D**) of 4–week (pre-symptomatic) and 8–week (symptomatic) cohorts in the pre-clinical trial. Data are expressed as mean ± standard error of the mean. C57BL/6 mice treated with control antibody (WT), *Mecp2*$^{T158A/y}$ mice treated with control antibody (P), *Mecp2*$^{T158A/y}$ mice treated with anti-Semaphorin4D antibody (T). * WT vs P, ~ WT vs T, ^ P vs T. *, ~, and ^ $p < 0.05$; **, ~~, and ^^ $p < 0.01$; ***, ~~~, and ^^^ $p < 0.001$.

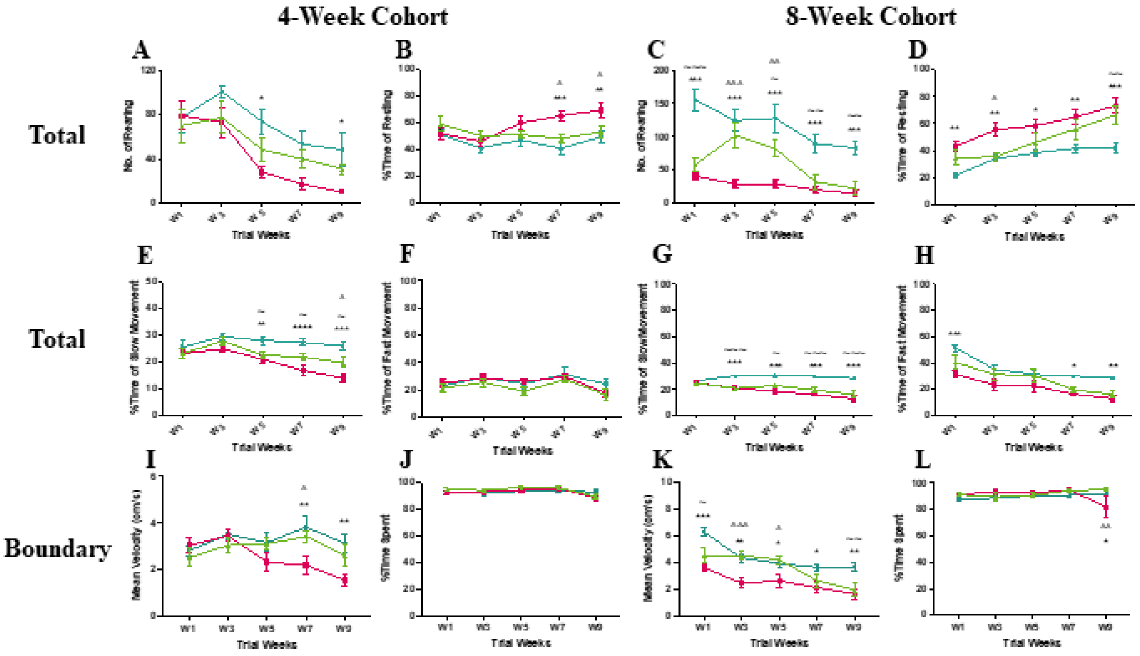

Supplementary Figure S2. Additional results of open field test of 4-week (pre-symptomatic) and 8-week (symptomatic) cohorts in the pre-clinical trial. Graphs display the analysis of total (**A–B, E–F**), and boundary (**I–J**) areas for the 4-week cohort, as well as total (**C–D, G–H**), and boundary (**K–L**) areas for the 8-week cohort. Data are expressed as mean ± standard error of the mean. C57BL/6 mice treated with control antibody (WT), *Mecp2*$^{T158A/y}$ mice treated with control antibody (P), *Mecp2*$^{T158A/y}$ mice treated with anti-Semaphorin4D antibody (T). * WT vs P, ~ WT vs T, ^ P vs T. *, ~, and ^ $p < 0.05$; **, ~~, and ^^ $p < 0.01$; ***, ~~~, and ^^^ $p < 0.001$.

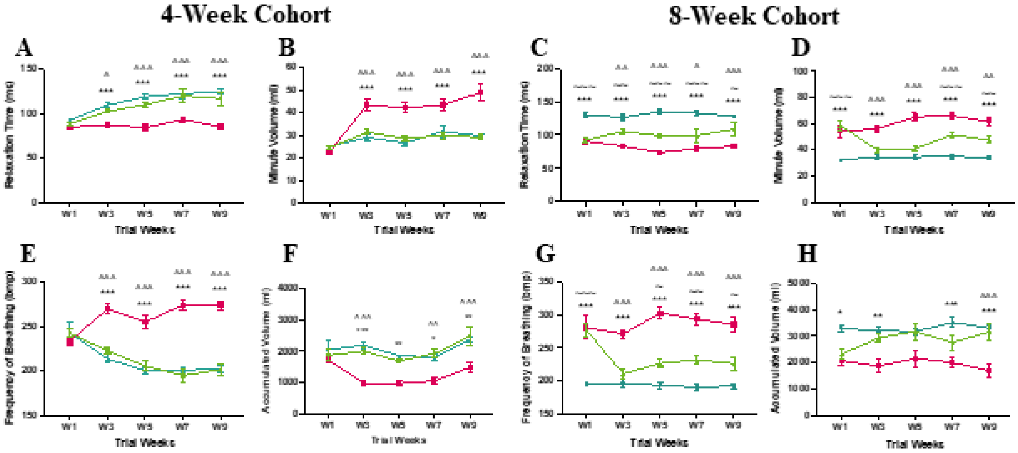

Supplementary Figure S3. Additional results of whole body plethysmography test of 4-week (pre-symptomatic) and 8-week (symptomatic) cohorts in the pre-clinical trial. Graphs demonstrate the relaxation time (**A** and **C**), the minute volume (**B** and **D**), the frequency of breading (**E** and **G**), and the accumulated volume (**F** and **H**) for the 4-week and the 8-week cohorts, respectively. Data are expressed as mean ± standard error of the mean.

C57BL/6 mice treated with control antibody (WT), *Mecp2*$^{T158A/y}$ mice treated with control antibody (P), *Mecp2*$^{T158A/y}$ mice treated with anti-Semaphorin4D antibody (T). * WT vs P, ~ WT vs T, ^ P vs T. *, ~, and ^ $p < 0.05$; **, ~~, and ^^ $p < 0.01$; ***, ~~~, and ^^^ $p < 0.001$.
